# Measuring the Immune Response to Tumor Vaccination

**DOI:** 10.1371/journal.pmed.0020360

**Published:** 2005-09-20

Tumor vaccination has perhaps been one of the most eagerly anticipated developments in cancer medicine. Research efforts in melanoma started with early unsuccessful attempts to induce a nonspecific immune response with Bacillé Calmette-Guerin (BCG). Subsequent vaccines combined BCG with autologous tumor cells or mixtures of allogeneic tumor cells, with varying responses. Most recently, however, work has focused on trying to produce a specific immune response using melanoma-derived peptide antigens. The antigens most commonly used are melanoma antigen recognized by T lymphocytes (MART-1)/Melan A, glycoprotein (gp) 100, and tyrosinase, all of which occur on both normal melanocyes and melanoma cells; randomized trials are currently under way on these vaccines.

However, the response of patients to these vaccines has been extremely variable and hard to predict. Ideally, researchers want to track antigen-specific T cells and measure their activation, but current assays are cumbersome and require large volumes of blood. Now, Mark Davis and colleagues from Stanford University and the University of Southern California present a high-throughput method for analyzing the response of patients to these vaccines, by means of an array that captures specific T cells, activates them, and then measures their response to activation. [Fig pmed-0020360-g001]

**Figure pmed-0020360-g001:**
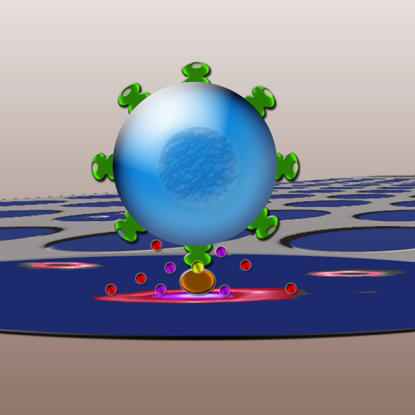
Catching antigen-specific T cells on a biological chip

The researchers demonstrated the usefulness of this approach by studying ten patients from a phase II trial of 60 patients who had stage IIC/III and IV melanoma and who had been vaccinated with a combination of MART-1, gp100, and tyrosinase. They describe the use of peptide major histocompatibility complex arrays, which immobilizes CD8T cells, activates them, and then measures the degree of activation by the secretion of cytokines. The major new technical development is the method of measuring the amounts of cytokines released by means of labeled antibodies also present on the array.

What they found was a startling diversity of responses to vaccination. However, one predictor of good clinical response to vaccination was strong secretion of both interferon-γ and tumor necrosis factor-α; four of the four patients with this pattern of secretion remained free of melanoma recurrence, whereas only two of the six patients in whom there were marked differences in the secretion of these two cytokines were free of recurrence.

Where does this paper leave the field of research on tumor vaccination? It provides a detailed snapshot of T cell responses in individual patients, and if these patterns of responses are substantiated in larger numbers of patients, it may well allow doctors to begin to understand who is likely to respond to one of these vaccines. What it does not do is explain why vaccine responses vary so much among patients; this is a far more complex question.

